# Role of personal care products as endocrine disruptors affecting reproductive age women

**DOI:** 10.3389/frph.2025.1514060

**Published:** 2025-07-11

**Authors:** Nitin Kalsi Rajashekara, Madhumitha Natarajan, Asha Srinivasan, Jovitha Babu, SubbaRao V. Madhunapantula, Bindu Jayshankar, Raghu Nataraj

**Affiliations:** ^1^ Division of Molecular Biology, School of Life Sciences, JSS Academy of Higher Education and Research, Mysuru, Karnataka, India; ^2^ Division of Cosmetic Science, School of Life Sciences, JSS Academy of Higher Education and Research, Mysuru, Karnataka, India; ^3^ Cellular and Molecular Biology, Centre of Excellence in Molecular Biology and Regenerative Medicine, Department of Biochemistry, JSS Medical College, JSS Academy of Higher Education and Research, Mysuru, Karnataka, India; ^4^ Department of Biotechnology, Sri Jayachamarajendra College of Engineering, JSS S&TU, Mysuru, Karnataka, India

**Keywords:** fertility, endocrine disruptors, personal care, lifestyle, menstrual cycle

## Abstract

The impact of endocrine-disrupting chemicals (EDCs) on public health is growing due to their wide-ranging consequence and likelihood of morbidity on human health. Humans are confronted with EDCs through their skin and drinks, as well as by inhaling. EDCs are extensively dispersed in the environment. EDCs have been shown to primarily influence puberty, the reproductive system, embryonic growth, the hypothalamus-pituitary-gonadal axis (HPG) neuroendocrine axis, and gender differentiation in the foetus, despite their capacity to influence a variety of hormone systems. Treatment for afflicted persons will benefit greatly from an understanding of the several ways that modifiable lifestyle circumstances connected to PCPS impede female infertility. The purpose of this review is to raise awareness of the hidden danger that environmental dyes (EDCs) pose to human health, particularly in terms of their detrimental effects on female reproductive health.

## 1 Introduction

An essential biological process for all living things is reproduction. Since the reproductive health of the parent species is essential to the continued existence of any species, any danger to reproductive health will provoke a robust response from the scientific community. Reproductive health indices have been reported to be declining globally during the past five to six decades, particularly in industrialized and affluent nations, due to factors associated with lifestyle that may be altered (1). The reproductive system is very susceptible to environmental stimuli. This is due to the fact that it requires energy expenditures, and it makes sense that, as in an organism, the physiological controls of the reproductive axis would be intimately correlated with nutritional condition (2). Scientific evidence has suggested that modifiable lifestyle factors (consumption of fat-rich diets, delayed childbearing/age of starting family, smoking, alcohol misuse, sexual behaviours, anxiety/depression, and perception/beliefs) play important roles in the general health and well-being of individuals, including fertility (3). Lifestyle variables are changeable behaviours and ways of living that have the potential to affect an individual's overall health and wellbeing, including fertility (4). They also have a significant role in determining an individual's exposure to the environment (5). A healthy lifestyle can be created by modifying behaviour and incorporating elements such as health consciousness, practical understanding of health sciences, motivation, and concern for taking action to protect and promote health.

**Table 1:** EDCs, their sources of exposure, possible mechanism of action, and their possible clinical outcomes in females.

Sl. No	Endocrine-disrupting chemical	Sources of exposure	Possible mechanism of action	Possible clinical condition
01	Bisphenol A (BPA)	Consumer products, including water bottles, food storage containers, dental sealants, and canned food lining (24)	Antagonist or Agonist of estrogen, involved in the estrogen-signalling pathway (23)	Lower ovarian reserve, Lower antral follicular count, PCOS (25–28)
02	Phthalates (PAEs)	Food packaging, toys, building materials, medical supplies, and personal care items, including cosmetics, lotion, and scents (32).	Antagonist or Agonist of estrogen, involved in the estrogen-signalling pathway, interferes with the feedback mechanism of the HPG axis (33)	Endometriosis, early pubertal onset, HPG axis dysregulations (34, 36, 38)
03	Polychlorinated Biphenyls (PCBs)	Paints, dyes, and carbonless copy paper, lubricants, and hydraulic fluids (39, 40)	Anti-androgenic, antagonist/agonist of estrogen, involved in the estrogen signalling pathway (39)	Endometriosis, breast cancer, and infertility (39, 40)
04	Dioxins	Numerous industrial operations using chlorine, for instance, burning garbage, bleaching paper and pulp, making chemicals, and producing various herbicides (43)	Dioxins act through the Arylhydrocarbon receptor (AhR), which is an intracellular protein that, in turn, dysregulates gene expression through the growth factor signal transduction pathway (41, 42).	Endometriosis, premature thelarche, breast cancer (39)
05	Pesticides	Direct exposure on the farm, contaminated food, air, etc (39)	Induction of aromatase, antiandrogenic, antiprogestin (39)	Infertility, precocious/early puberty, risk of breast cancer, PCOS, infertility (39, 47–49)
06	Metals in PCPs	Cosmetics (51, 52)	Antagonist/agonist of estrogen, involved in the estrogen signalling pathway, involved in the steroidogenic pathway, and Endoplasmic Reticulum Stress (ERS) related IRE1*α*-JNK signalling pathway, ERK1/2 pathway (53, 55, 57)	Irregular menstrual cycle, defective pubertal onset, dysmenorrhoea, infertility (52, 54, 56)
07	Triclosan (TCS)	Personal care products and household products (58)	Interrupt the estrogen signalling pathway (59)	Menstrual irregularity, abnormal foetal development, infertility (58, 59)

A subset of self-care items known as personal care products (PCPs) is often used for grooming, cleaning, personal hygiene, and beautifying. Products that readily expose individuals include those for hair and skin care, baby care, UV blocking creams, facial cleansers, insect repellents, perfumes, scents, soap, detergents, shampoos, conditioners, toothpaste, and more. The frequency of PCP utilization is a highly varied personal choice that is influenced by lifestyle circumstances and socioeconomic status (6). Furthermore, one of the primary contributors of contaminants that are surfacing in the environment includes PCPs. PCPs prevail in human bodies at all phases of life, including intrauterine development. Inhalation, cutaneous interaction, ingestion, and absorption are the direct modes of exposure; product use and environmental contamination are the direct pathways (7). The most prevalent sort of merchandise found in homes and public areas in PCPs. Globally, between 30 and 40 percent of dermatologist prescriptions comprise at least one PCP, and a single person utilizes at least two PCPs in a 24 h period (8, 9). The total mass loading of PCPs in the Human Province of Southern China was 506.35 mg/d/1,000 people, which contributed to the overall emission of 357.56 mg/d/1,000 people (10). A variety of compounds are released by PCPs. For instance, the only products that emit 49.25 and 9,574 µg of siloxanes per individual per day are shampoo and shower gel. The air was found to be contaminated with decamethylcyclopentasiloxane (D5) and dodecamethylcyclohexasiloxane (D6), with per capita emission levels of 8.33 and 6,109 µg/day, respectively (11). Likewise, phthalates from PCPs contaminate indoor air (12). Monoethanolamine and diethanolamine are commonly found in cleaners, shampoos, hair dyes, and detergents (13). The TESIE study identified phthalates in almost all hand wipes and dust samples, and their metabolites were detected in all children's urine samples, confirming their ubiquitous exposure (14). Several of these compounds are referred to as endocrine-disrupting chemicals (EDCs), and because of their potential for morbidity and wide-ranging impacts on human health, public health is now starting to place greater attention on EDCs. EDCs are widely distributed in the environment, and humans become susceptible to them through their skin, in their consumption of food and drink, and through their respiration. EDCs have been shown to primarily impact puberty, embryonic development, the reproductive system, and sex differentiation in the fetus, despite their capacity to influence a variety of hormone systems. Consequently, the most plausible explanation for their primary mode of action is that they interfere with sex steroid hormones (15). Comprehending the diverse mechanisms through which modifiable lifestyle-associated PCPs hinder female infertility will significantly aid in the treatment of affected individuals. This review attempts to shed light on the unnoticed harm that EDCs cause to human health, particularly as endocrine disruptors hampering female reproductive health.

## 2 Endocrine-disrupting chemicals (EDCs)

**Figure 1:**
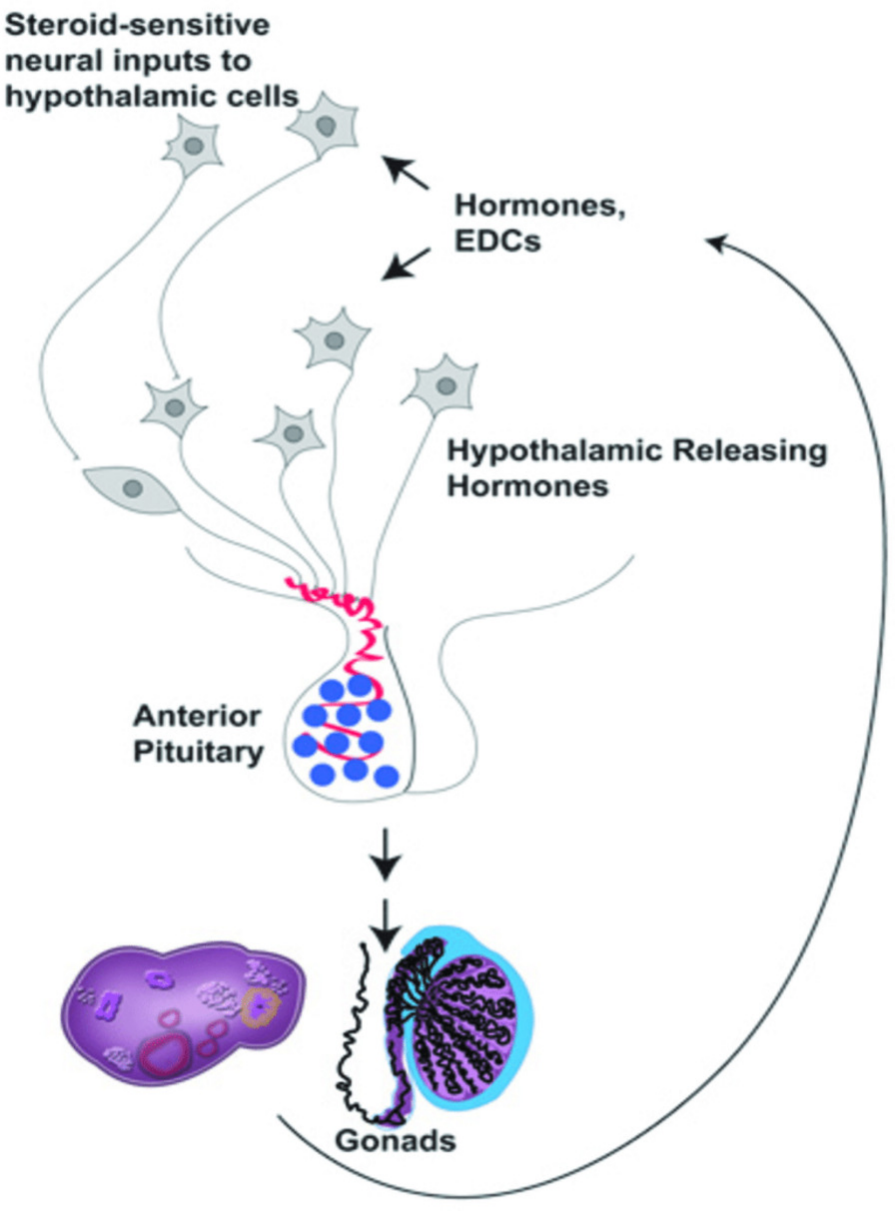
The hypothalamic-pituitary-gonadal (HPG) axis (22).

“An exogenous (non-natural) chemical, or a mixture of chemicals, that interferes with any aspect of hormone action” is how the Endocrine Society defines EDCs. By functioning as hormone antagonists, imitating hormones, interfering with hormone production or breakdown, changing the process by which hormone receptors develop, or altering hormone binding, these substances affect the body's hormonal balance in a wide range of ways (15). The manufacture and consumption of man-made chemicals such as flame retardants, chemical pesticides, plastics and plasticizing agents, electronic waste materials, food additives, metallic substances, and personal care products are the main sources of environmental EDCs. These EDCs have the potential to throw off the hormonal balance, which can result in a host of health problems, including immunological system dysfunction, growth abnormalities, and neurodevelopmental delays in children, abnormalities in the reproductive and developmental processes, and hormone-sensitive cancers. It is recognized that EDCs modify the metabolic balance via several methods, such as changes to peroxisome proliferator-modulated pathways (16), adipogenesis (17), pancreatic β-cell function (18–20), and hypothalamic neuropeptides (21, 22). The majority of data on the direct impact of EDCs on fertility is gathered from studies done using animals and *in vitro*. Although several studies have demonstrated a direct correlation between EDCs and infertility parameters, other studies have associated them with PCOS, a complex endocrinopathy that results in insulin resistance and infertility, concurrently damaging the HPG neuroendocrine axis (Figure 1). The current review discusses how PCPs, as endocrine disruptors, affect female reproductive outcomes.

### 2.1 Bisphenol A (BPA)

**Figure 2:**
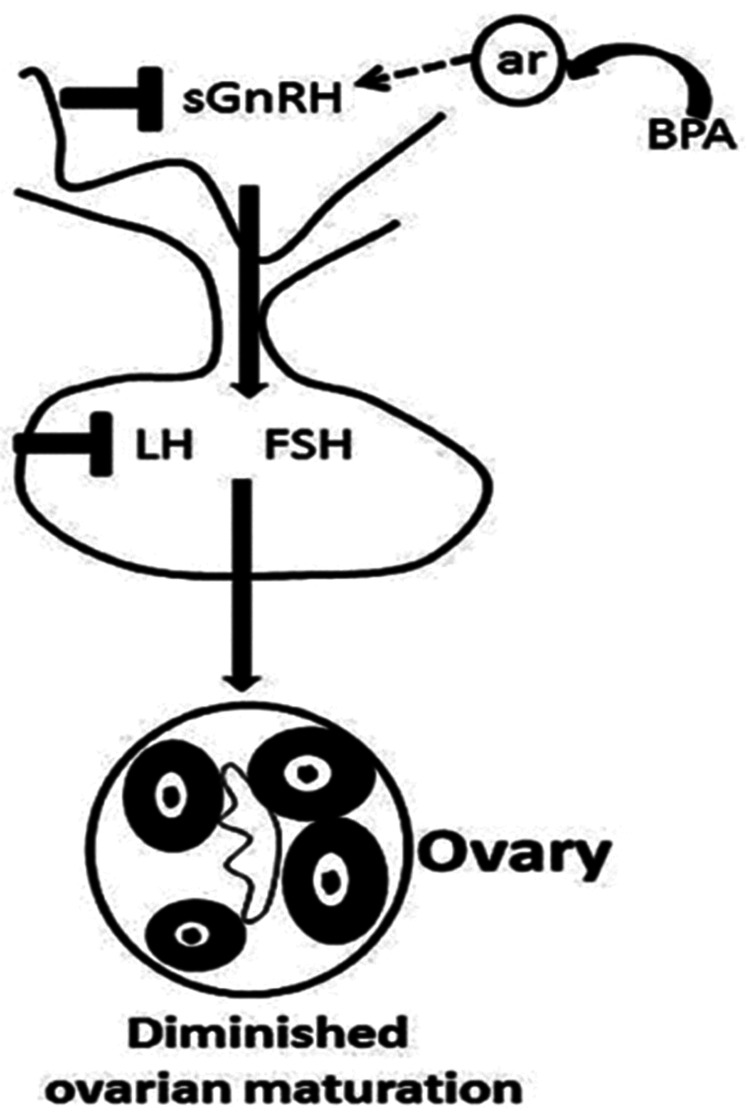
BPA reduced ovarian maturation by affecting the HPG axis (31).

Found in a variety of plastics, food containers, and receipts. Bisphenol A (BPA) is a chemical that has been widely utilized in the production of different plastics and epoxy resins since the 1960s. Consumer products, including water bottles, food storage containers, dental sealants, and canned food lining, are common places to find it. The ability of BPA to harden polymers and increase the toughness of various materials is well documented. However, concerns about the potential health effects of BPA exposure have been highlighted. Because it can interfere with the body's hormonal system, BPA is regarded as an endocrine disruptor. The BPA is a xenoestrogen that interacts with the estrogen receptor and acts as an antagonist or agonist of estrogen, involved in the estrogen-signalling pathway and disrupts the endocrine system (Table 1) (23). Numerous health concerns, such as those pertaining to reproduction, development, and an elevated risk of certain ailments, have been associated with it (24). BPA was discovered to be linked to a lower ovarian reserve in previous investigations, along with (25) lower antral follicle count (26) and PCOS (27, 28) in infertile women. Additionally, another study elsewhere has demonstrated that higher quartiles of urine BPA content are linked to a higher risk of implantation failure (29). A case study report on BPA/BPB has pointed out the definite association between endometriosis prevalence and BPA and/or BPB levels in the blood (30). Another case-control study conducted in China on BPA implies that BPA may impact ovarian follicles in PCOS women, hence lowering ovarian reserve (25). Additionally, BPA could reduce ovarian maturation, and this reduction could be recovered after BPA treatment withdrawal (Figure 2) (31). The United Kingdom case-control studies have identified BPA as an endocrine disruptor that may play an essential role in the pathophysiology of PCOS, as being identified by a statistically significant positive association between androgens and BPA, along with the greater BPA levels in PCOS women compared to controls (27).

**Figure 3:**
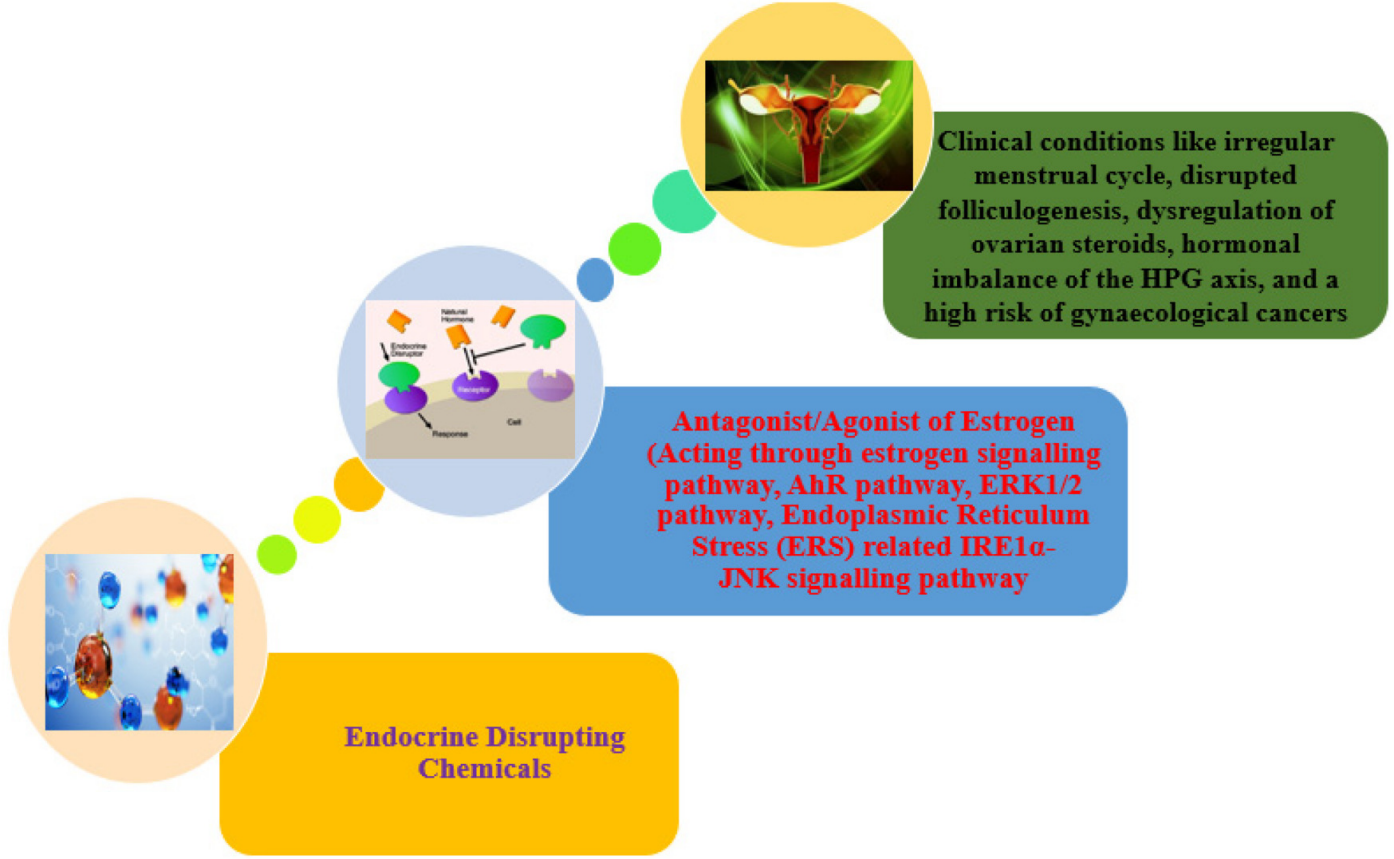
Overview of EDCs causing female reproductive disorders.

### 2.2 Phthalates (PAEs)

A class of chemical substances known as phthalates is frequently added to plastic as plasticizers-substance that give polymers more flexibility, transparency, resilience, and lifespan. They are frequently present in an extensive variety of commodities, including food packaging, toys, building materials, medical supplies, and personal care items, including cosmetics, lotion, and scents. Urinary metabolite concentrations were demonstrated to have a negative correlation with serum inhibin B levels, indicating that phthalates have a deleterious influence on the growth of antral follicles (Table 1) (32). Phthalates act as the xenohormone for both androgen and estrogen, which blocks the corresponding receptors, which interferes with the feedback mechanism of the HPG axis (33). Korea case-control Research phthalate study has demonstrated that individuals with advanced-stage endometriosis had considerably higher plasma levels of monoethylhexyl phthalate and DEHP (34). Particularly, phthalates may have detrimental effects on reproductive and developmental health, according to a specific study. As a precaution, some people opt to minimize their exposure to products that contain phthalates, especially pregnant women and young children who are more susceptible than other populations. USA Research using case-control phthalate suggests that phthalates might alter a woman of reproductive age and risk of hormone-mediated illness (35). Thailand cross-sectional research phthalates, increased concentration of mono-ethyl-phthalates (MEP) was associated with girls who have reached puberty early (36). USA cohort study Longitudinal phthalates Research indicates that at some crucial stages of *in utero* development, female reproductive development may be more susceptible to the negative effects of phthalate or BPA exposure (37). As an essential endocrine axis that regulates the reproductive system, whether dysfunction of the hypothalamus-pituitary-gonadal (HPG) axis is involved in reproductive toxicity mediated by environmental endocrine disruptors, PAEs has become a hot topic of widespread concern (Figure 3) (38).

### 2.3 Polychlorinated biphenyls (PCBs)

The stable, non-flammable, and electrically insulating properties of polychlorinated biphenyls, or PCBs, are highly valued in synthetic organic molecules. To create PCBs, a biphenyl molecule, a substance with two benzene rings linked to chlorine atoms. PCBs were commonly discovered in electrical equipment, including lubricants and hydraulic fluids, as well as transformers and capacitors. They were also used in a number of industrial operations, including the creation of paints, dyes, and carbonless copy paper. The PCBs act as agonists or antagonists of estrogen, and also show antiandrogenic activity (Table 1) (39). In a case-control study, France found that persons with deep infiltrating endometriosis (DIE) had greater internal exposure levels of various PCBs, BFRs, OCPs, and dioxins in adipose tissue, particularly in severe instances of endometriosis (Stages III–IV) (40).

### 2.4 Dioxins

Dioxins are a category of extremely toxic chemicals that are persistent environmental pollutants. They belong to the categories of chemical compounds known as polychlorinated dibenzofurans (PCDFs) and polychlorinated dibenzo-p-dioxin (TCDD). Numerous industrial operations using chlorine, for instance, burning garbage, bleaching paper and pulp, making chemicals, and producing various herbicides, accidentally result in the production of dioxins. Dioxins may reach the food chain through polluted soil, water, or air and gather in the animal's fatty tissues. Humans are most exposed through the consumption of contaminated food, particularly animal products like meat, dairy, and seafood. Dioxins act through the Arylhydrocarbon receptor (AhR), which is an intracellular protein that, in turn, dysregulates gene expression through the growth factor signal transduction pathway (41, 42). In the Spanish case-control study, individuals with DIE had substantially higher levels of dioxins and PCBs in their adipose tissue compared to the control group (*p* < 0.05) (Table 1) (43).

### 2.5 Pesticides

Chemicals identified as pesticides are used to regulate or eliminate the presence of pests that can destroy crops, kill livestock, or cause additional issues. Many pesticides can interact with the endocrine systems of both humans and animals because they are endocrine disruptors. Hormones, which are essential for metabolism, growth, development, and reproduction, are regulated by the endocrine system. Certain pesticides can alter, suppress, or mimic the body's hormones from within, which may cause problems with the endocrine system. Numerous health issues, including abnormal growth, trouble becoming pregnant, and modification to the usual operation of the hormone-regulated organs, might result from this interference. Sources of drinking water have been found to contain pesticides, some of which are known to be harmful to reproduction. Most pesticides act as antiandrogenic, antiprogestin agents. They also act as aromatase inducers and reduce the production of insulin-like growth factors (39). For instance, reduced sperm counts and negative pregnancy results in both humans and non-human primates are linked to pesticide exposure (Table 1) (44–46). According to an investigation, inadequate outcomes from embryological intracytoplasmic sperm injection were connected with high quantities of PCB and pesticides in the follicular fluid (47). According to another study, women with PCOS had greater blood concentration of phthalate metabolism and perfluorinated chemicals (48). DDT may have a part in the pathophysiology of PCOS in Chinese women with the condition due to its association with altered hormone levels. An early menopausal onset was linked to EDCs. According to a large cross-sectional study that included over 30,000 women (49). Additionally, they found 15 EDCs (comprising 3 pesticides, 2 phthalates, 1 furan, and 9 PCBs) that required additional assessment to rule out any potential detrimental effects on ovarian function (50).

### 2.6 Metals in PCPs

Chemical bleaching chemicals and possibly hazardous ingredients are found in skin-lightening cosmetics, according to a comprehensive literature review (Table 1) (51). According to 25 research studies that the group reviewed, 12 of the research studies examined mercury alone, while 13 of them examined mercury in conjunction with other widely used active ingredients for skin lightening (such as betamethasone, clobetasol propionate, kojic acid, hydroquinone, and corticosteroids) or trace elements (such as bismuth, cadmium, chromium, cobalt, copper, lead, iron, nickel, manganese, palladium, thallium, titanium, titanium dioxide, zinc, and arsenic). Mercury (Hg) has demonstrated an increased incidence of irregular menstrual periods (52). Hg acts as an antagonist or an agonist of estrogen, interfering with the estrogen-signalling pathway (53). Lead (Pb) is associated with a delay in pubertal development and growth of girls (54). A study conducted on rats shows a downregulation of steroidogenic genes such as STAR, CYP17A1, and HSD3B1, upregulation of FSHR and CYP19A1 upon Pb exposure. The study has also noticed the stimulation of the apoptotic pathway along with the stimulation of Endoplasmic Reticulum Stress (ERS) related IRE1α-JNK signalling pathway members, which leads to the dysregulation of the HPG axis hormonal profile, folliculogenesis, and delayed pubertal onset upon juvenile exposure to Pb (55). Cadmium (Cd) has been reported to be associated with abnormal menstrual cycle, dysmenorrhoea in unmarried women, and sterility in married women (56). Studies have observed a decreased folliculogenesis upon Cd exposure in animals. It is well demonstrated that Cd exposure leads to an accumulation of hydrogen peroxide with a decrease in antioxidant enzymes in the ovaries, which in turn causes the apoptosis of ovarian cells, and results in the downregulation of ovarian steroids. It is also shown that Cd exposure causes the rapid activation of the ERK1/2 pathway and thereby interferes with the estrogen signalling pathway (57).

### 2.7 Triclosan (TCS)

2,4,4-trichloro-2′-hydroxydiphenyl ether, commonly known as triclosan (TCS), is an antibacterial agent used in personal care and household items that has brought attention as it affects female reproductive health. Emerging research on TCS concerning female reproductive health has shown its effect on endocrine disruption, which causes the hormonal dysregulation resulting in irregular menstruation, infertility, and abnormalities in the development of the foetus (Table 1) (58). Studies have shown that the TCA will bind to the estrogen receptor, which may lead to irregular menses and hormonal disruption (59). Animal studies have also demonstrated the bioaccumulation of TCA in the ovarian and uterine tissues, which leads to a compromise in the reproductive function (60). Further research is recommended to unveil the major effects of TCA as an endocrine disruptor.

## 3 Conclusion

**“**Endocrine disruptors” are compounds that have the ability to interfere with the endocrine system, an intricate web of hormones and glands that control a wide range of bodily physiological functions. These disturbances can mimic, hinder, or inhibit endogenous hormone generation, binding, action, metabolism, release, transport, or excretion. As a result, they may disrupt the normal function of the endocrine system, which may have detrimental effects on growth and well-being. Endocrine disruptor exposure has been linked to several health issues, including immune system malfunction, abnormal development, reproductive abnormalities, and an elevated risk of multiple cancers. It is believed that young toddlers, neonates, and pregnant women are most vulnerable to the effects of these disruptors. The hazards and exposure levels to parabens and bisphenols associated with Indian women's consumption of personal care products (PCPs) are not well recognized. It is imperative that individuals follow safety protocols while handling or applying pesticides and be aware of the potential risks associated with pesticide exposure. Furthermore, consuming organic food, adopting integrated pest management strategies, and supporting sustainable agriculture practices can all help reduce exposure to chemicals that have endocrine-disrupting properties. Global regulatory agencies, including the Environmental Protection Agency (EPA) in the US and the European Food Safety Authority (EFSA) in Europe, analyse the potential of pesticides to interfere with the endocrine system before approving their usage. A lot of work goes into determining safe exposure thresholds and limiting or outlawing the use of pesticides that seriously endanger human health. To reduce the effects of endocrine disruptors on the environment and public health, regulatory organizations from all over the world are trying to discover and control their use. The possible dangers are consequence of exposure to these compounds are still being investigated. Additionally, people can lessen their exposure by using glass or stainless-steel containers instead of plastic ones, choosing items that are labelled as “BPA-free” or “Phthalates-free”, and paying attention to the products they use regularly. PCBs may still be present in some older materials and equipment despite the bans and regulations, creating ongoing problems for public health and environmental management. Concerns over PCB contamination persist, and clean-up initiatives are being undertaken in affected areas. Cleansing techniques include the containment or removal of polluted soil, water, and silt. Effective disposal of products containing PCBs is an essential aspect of remediation. Studies have shown that EDCs, such as phthalates, bisphenols, and parabens, may have negative health impacts on people, yet these chemicals are widely regarded as harmless and are present in a variety of goods. The fundamental reason is that the applicable regulation requires companies to keep trace quantities of suspicious chemical substances on reserve. That being said, it's crucial to consider the possibility of combination effects (synergism, additivity, inhibition) when there are several endocrine disruptors. Considering that the actual chemical combination cannot be safely exposed to at theoretically acceptable concentrations of separate substances. The majority of research on the negative effects of EDCs comes from studies conducted on animals. Along with the manufacturing and dispersion of EDCs into the surroundings, potable water supplies, and eventually the food chain, global industrialization is contributing to the sharp increase in the prevalence of numerous diseases, most notably HPG neuroendocrine axis disruption associated with reproductive endocrine disorders. The anterior pituitary gonadotrophs are the specific organs targeted by the release of GnRH from hypothalamic neurons to produce/secrete LH and FSH. From that point, gonadal steroidogenesis and gametogenesis are triggered by these circulating gonadotropins acting on receptors in the gonad, ovary, or testis. In addition to acting on peripheral targets, steroid hormones in the bloodstream also feedback on steroid-sensitive brain neurons, which provide inputs to the hypothalamus. Henceforth, EDC exposure during development can disrupt HPG systems by interfering with any unexplored mechanisms. Consequently, additional investigation is required to identify the EDC threshold concentrations in human matrices below which negative effects do not manifest. Comprehensive, systematic epidemiological studies that consider the combination and low-dose effects of EDCs are required.
